# Complications and outcomes following surgical management of common calcaneal tendon pathology in 80 dogs

**DOI:** 10.1111/vsu.70053

**Published:** 2025-11-03

**Authors:** Sebastian Wylie, Francesco Piana, Philip Montgomery, Sarah Girling, Luca Vezzoni, Richard Meeson, Alex Belch, Kevin Parsons

**Affiliations:** ^1^ University of Bristol Bristol UK; ^2^ Langford Veterinary Services, Small Animal Hospital Bristol UK; ^3^ Wear Referrals Bradbury UK; ^4^ Fitzpatrick Referrals Godalming UK; ^5^ Clinica Veterinaria Vezzoni Cremona Italy; ^6^ Royal Veterinary College North Mymms UK

## Abstract

**Objective:**

To report the complications and outcomes following surgical management of common calcaneal tendon (CCT) pathology in dogs.

**Study design:**

Retrospective cohort study.

**Sample population:**

A total of 80 dogs with CCT pathology underwent 89 surgeries.

**Methods:**

Retrospective data were reviewed from five veterinary referral centers for dogs with CCT pathology that underwent surgical treatment (January 2011 to December 2021). Clients completed a Liverpool Osteoarthritis in Dogs (LOAD) questionnaire to assess long‐term outcomes.

**Results:**

Tendon repair with tarsocrural immobilization was performed in 46/89 limbs (51.7%), with three‐loop pulley the most common suture pattern, used in 19/46 tendon repairs (41.3%). Pantarsal arthrodesis was performed in 32/89 limbs (36%) and temporary tarsocrural immobilization without tendon repair in 11/89 limbs (12.3%). Median time from surgery to final follow‐up at the referral center was 10 weeks (range: 6–256 weeks). There was no difference in complication rate between tendon repair (56.5%) and pantarsal arthrodesis (42.8%) (*p* = .543). Tendon repair with tarsocrural immobilization had a significantly higher catastrophic complication rate (26.1%) than pantarsal arthrodesis (0%) (*p* = .005). A total of 23 LOAD questionnaires were returned. There was no difference in mildly affected dogs between the three surgical groups (*p* = .493).

**Conclusion:**

Pantarsal arthrodesis and CCT repair surgeries had comparable short‐term outcomes and complication rates. However, there is a greater risk of complications requiring revision surgery following temporary tarsocrural immobilization, with or without tendon repair, compared to pantarsal arthrodesis.

**Clinical significance:**

The increased risk of revision surgery should be discussed with owners, considering the potential financial and treatment implications for their dogs.

**Abbreviations**
CCT: Common calcaneal tendon
CT screws: calcaneotibialscrews
CT: combined tendon
GT: gastrocnemius
LOAD: Liverpool Osteoarthritisin dogs
PDS: polydioxanone
SDFT: superficial digital flexor tendon
TESF: transarticularexternal skeletal fixators

## 1 INTRODUCTION

The common calcaneal tendon (CCT), or Achilles tendon, is composed of three separate tendons: the tendon of the gastrocnemius (GT), the superficial digital flexor tendon (SDFT) and the combined tendon of the gracilis, semitendinosus and biceps femoris (CT).^1^, ^2^ Pathology of the CCT is often associated with chronic degeneration, with most dogs presenting acutely lame following normal exercise. Less commonly an acute traumatic injury is observed.^3^ Typically, the GT and CT are affected with the SDFT spared, resulting in a characteristic plantigrade stance with flexion of the digits, and swelling over the CCT.^3^, ^4^

Plantigrade stance is debilitating to dogs, and surgery is typically recommended to return them to a digitigrade position either through repair of the CCT, temporary tarsocrural immobilization or pantarsal arthrodesis. The decision as to which surgery to perform is often based on individual surgeons' experience and the results of a discussion with the owner pertaining to the risks and complications of each procedure, as well as the expected outcome.

Tendon repair typically involves debridement of fibrous scar tissue (where appropriate), followed by application of a tendon suture, with three‐loop pulley and Kessler locking loop described.^5^ The function of these sutures is to oppose the tendon edges and to resist gap formation. While cadaveric studies offer conflicting conclusions, retrospective clinical studies have found no difference in outcome when comparing suture patterns.^6^, ^7^, ^8^, ^9^, ^10^, ^11^ Further additions to sutures have also been suggested. These include synthetic implants, platelet‐rich plasma, stem cells and flexor digitorum lateralis grafts.^12^, ^13^, ^14^, ^15^, ^16^, ^17^, ^18^

Tendon healing is particularly vulnerable to gap elongation in the first 3 weeks and therefore tarsocrural immobilization is recommended. Tarsocrural immobilization can be achieved through the application of transarticular external skeletal fixators (TESF), calcaneotibial screws (CT screws), transarticular calcaneotibial plates or splints and casts.^4^, ^19^, ^20^ These methods can be used alone or in combination. Typically, the majority of complications reported following CCT repair come from the method of immobilization rather than the repair itself.^4^

An alternative to tendon repair is pantarsal arthrodesis, as a salvage procedure if the tendon repair fails or as a first line treatment for CCT rupture. Pantarsal arthrodesis has a reported complication rate ranging from 30% to 70%, with implant failure and plantar necrosis reported as the most common major complications; however, some surgeons advocate a straight to arthrodesis approach.^21^, ^22^, ^23^

We hypothesized that dogs undergoing pantarsal arthrodesis as a primary treatment for CCT pathology would have improved outcomes and lower complication rates compared to dogs treated with tendon repair or temporary tarsocrural immobilization. It is hoped that the findings will provide further information to help guide clinicians' treatment and discussions with owners when deciding how to treat CCT pathology in dogs.

## 2 MATERIALS AND METHODS

Medical records of dogs that underwent surgical management for CCT tendinopathy between January 2011 and December 2021 at five referral veterinary hospitals were reviewed. Cases were excluded if they did not have a minimum of 6 weeks in‐clinic follow‐up, were missing signalment data, or if a previous surgery to treat CCT pathology had been performed. Recorded data included signalment, presenting clinical signs, duration of clinical signs, type of injury (acute or chronic if clinical signs had been present for less than or over 3 weeks prior to presentation), preoperative imaging, surgical management technique, suture material used for tendon repair, tarsocrural immobilization method, postoperative management and postoperative complications.

Surgeries were categorized based on the initial surgical treatment: tendon repair with temporary tarsocrural immobilization, temporary tarsocrural immobilization without tendon repair or pantarsal arthrodesis. Subsequent surgical interventions were not used to reclassify cases.

Complications and short‐term outcomes were categorized as described by Cook et al.^24^ Complications were categorized as minor if they did not require further intervention to resolve. Major type 1 complications required surgical management to resolve but did not result in salvage procedures or long‐term functional loss. Major type 2 complications required medical treatment to resolve. Catastrophic complications were those that resulted in permanent unacceptable function, death, euthanasia, or required repeat tendon repair, pantarsal arthrodesis, or amputation following an initial surgery. Dogs that underwent staged bilateral surgery were considered as two separate surgeries. Postoperative complications were categorized by time: perioperative (0–3 months), short‐term (3–6 months), mid‐term (6–12 months) or long‐term (>1 year). At the final follow‐up examination outcomes were categorized as either full function, acceptable function or unacceptable function.^24^ Outcomes were categorized according to the initial surgery performed.

Long‐term outcomes were assessed through clients completing the Liverpool Osteoarthritis in Dogs (LOAD) questionnaire, a validated owner‐completed instrument designed to assess canine mobility and chronic pain associated with orthopedic disease. The LOAD score has been shown to correlate with force plate gait analysis and is considered a reliable measure of functional outcome in dogs with musculoskeletal conditions.^25^ Clients were contacted via email or telephone. Cases with scores 0–10 were categorized as mildly affected, 11–20 as moderately affected, 21–30 as severely affected and, >30 as extremely affected.^26^

### 2.1 Statistical analysis

Statistical analysis was performed using software (SPSS IBM Statistics version 20; Graph Pad quick calculations [http://www.graphpad.com/quickcalcs]). Descriptive statistics were performed and reported as mean (±SD) or median and ranges for parametric and non‐parametric data, respectively. Where appropriate, chi‐squared or Fisher's exact test were used to compare categorical data. Significance was set at *p* < .05.

## 3 RESULTS

### 3.1 Signalment, presentation and preoperative imaging

A total of 80 dogs met inclusion criteria with nine dogs undergoing staged bilateral surgery, resulting in 89 surgeries (Table 1). There were 26 breeds represented, including Labrador Retrievers with 26/80 dogs (32.5%) and Springer Spaniels with 5/80 dogs (6.3%). Female neutered dogs represented 34/80 dogs (42.5%) and male neutered 25/80 dogs (31.3%). The median age was 7 years (range: 0.66–13.1 years) and median weight was 30.0 kg (range: 8.8–70.0 kg).

**TABLE 1:** Description of presenting clinical signs, complication category and overall short‐term outcomes. Multiple complications occurred in two limbs in the tarsocrual immobilization with tendon repair group, two limbs in the pantarsal arthrodesis group and one limb in the tendon repair without tarsocrural immobilization group.

	Tarsocrural immobilization with tendon repair	Pantarsal Arthrodesis	Tarsocrural immobilization without tendon repair	Total limbs	*p*‐value
Number of limbs	46 (51.6%)	32 (35.9%)	11 (12.3%)	89 (100%)	
Breeds					
Labrador	12 (26.1%)	11 (34.4%)	4 (36.4%)	27 (30.3%)	.661
Springer Spaniel	2 (4.3%)	3 (9.4%)	0 (0%)	5 (5.6%)	.439
Doberman Pinscher	1 (2.2%)	3 (9.4%)	0 (0%)	4 (4.5%)	.238
Cross breeds	14 (30.4%)	6 (18.8%)	1 (9.1%)	21 (23.6%)	.235
Other breeds	17 (37.0%)	9 (28.1%)	6 (54.5%)	32 (36.0%)	.283
Clinical signs					
Tendon swelling	33 (71.7%)	19 (59.4%)	8 (72.7%)	60 (67.4%)	.478
Plantigrade stance	37 (80.4%)	17 (53.1%)	5 (45.5%)	59 (66.3%)	.004
Digit flexion	18 (39.1%)	16 (50.0%)	3 (27.3%)	37 (41.6%)	.373
Acute	17 (37.0%)	9 (28.1%)	7 (63.6%)	33 (37.1%)	.109
Chronic	29 (63.0%)	23 (71.9%)	4 (36.4%)	56 (62.9%)	.109
Complications					
Complication rate	26 (56.5%)	15 (46.9%)	5 (45.5%)	46 (51.7%)	.898
Minor	2 (4.3%)	2 (6.3%)	2 (18.1%)	6 (6.7%)	.257
Major 1	3 (6.5%)	4 (12.5%)	0 (0%)	7 (7.9%)	.368
Major 2	11 (23.9%)	11 (34.4%)	2 (18.1%)	24 (26.9%)	.463
Catastrophic	12 (26.1%)	0 (0%)	2 (18.1%)	14 (15.7%)	.008
Short‐term outcomes					
Full function	7 (15.2%)	12 (37.5%)	2 (18.1%)	21 (23.6%)	.067
Acceptable function	23 (50.0%)	14 (43.8%)	6 (54.5%)	3 (48.3%)	.783
Unacceptable function	14 (30.4%)	5 (15.6%)	2 (18.1%)	21 (23.6%)	.287
Unknown	2 (4.3%)	1 (3.1%)	1 (9.1%)	4 (4.5%)	.711

The median interval between first clinical signs and surgery was 38 days (range: 0–1033 days). The most common presenting clinical signs were CCT thickening/swelling in 60/89 limbs (67.4%), plantigrade stance in 59/89 limbs (66.3%), and digit flexion in 37/89 limbs (41.6%). Preoperative lameness score was recorded in 73/89 limbs (82%), median preoperative lameness score was 3/5 (range: 1/5–5/5) in all three surgical groups.

An acute trauma with tendon rupture occurred in 16/89 limbs (18%), 10 of these limbs had wounds over the CCT or hock on presentation. In this cohort, 12/16 limbs underwent tarsocrural immobilization with tendon repair, 1/16 underwent tarsocrural immobilization without tendon repair and 3/16 underwent pantarsal arthrodesis.

Preoperative imaging was performed in 80 limbs: radiographs in 64/80 (80%), ultrasound in 31/80 (38.8%) and computed tomography (CT) scans in 3/80 (3.8%). A total of 16 limbs (20%) had both preoperative radiographs and ultrasound.

### 3.2 Surgery—Joint immobilization with tendon repair

Tendon repair with joint immobilization was performed in 46/89 limbs (51.7%), the most commonly used method of tendon repair was three‐loop pulley sutures with polydioxanone (PDS) (Table 2). In one of the tendon repair cases a Vetlig implant (Vetlig Limited, Peterborough, UK) was used alongside a three‐loop pulley suture. Following tendon repair surgery the tarsocrural joint was immobilized using: a calcaneotibial screw and cast in 18/46 limbs (39.1%), TESF in 20/46 limbs (43.5%), transarticular plate in 5/46 limbs (10.9%), cast alone in 2/46 limbs (4.3%) and calcaneotibial screw alone in one limb (2.2%). The transarticular plates consisted of three LCP plates (all 3.5‐mm) and two locking T‐plates (one 3.5‐mm and one 2.4/2.7‐mm).

**TABLE 2:** Summary of the tendon repair method used and methods of tarsocrural immobilization for the tendon repair group. Multiple complications occurred in two limbs in the three‐loop pulley group.

	Three‐loop pulley	Locking loop	Unknown pattern	Three‐loop pulley and locking loop	Total	*p*‐value
Number of limbs	19 (41.3%)	12 (26.1%)	11 (23.9%)	4 (8.7%)	46 (100%)	
Suture material						
PDS	13 (68.4%)	4 (33.3%)	2 (18.1%)	0 (0%)	19 (41.3%)	
Prolene	2 (10.5%)	6 (50.0%)	6 (54.5%)	4 (100%)	18 (39.1%)	
PDS and Prolene	0 (0%)	1 (8.3%)	1 (9.1%)	0 (0%)	2 (4.3%)	
Biosyn and Prolene	0 (0%)	1 (8.3%)	1 (9.1%)	0 (0%)	2 (4.3%)	
Biosyn	0 (0%)	0 (0%)	1 (9.1%)	0 (0%)	1 (2.1%)	
Unknown	4 (21.1%)	0 (0%)	0 (0%)	0 (0%)	4 (8.7%)	
Tarsocrural immobilization method						
CT screw	1 (5.2%)	0 (0%)	0 (0%)	0 (0%)	1 (2.1%)	
CT screw with cast	12 (63.1%)	4 (33.3%)	1 (9.1%)	1 (25.0%)	18 (39.1%)	
TESF	4 (21.1%)	4 (33.3%)	9 (81.8%)	3 (75.0%)	20 (43.4%)	
Transarticular plate	1 (5.3%)	3 (25.0%)	1 (9.1%)	0 (0%)	5 (10.9%)	
Cast	1 (5.3%)	1 (8.3%)	0 (0%)	0 (0%)	2 (4.3%)	
Complications						
Complication rate	9 (47.4%)	6 (50.0%)	9 (81.8%)	2 (50%)	26 (56.5%)	.285
Minor	1 (5.3%)	0 (0%)	1 (9.1%)	0 (0%)	1 (2.2%)	.715
Major type 1	2 (10.5%)	1 (8.3%)	0 (0%)	0 (0%)	3 (6.5%)	.657
Major type 2	3 (15.7%)	3 (25.0%)	3 (27.2%)	2 (50.0%)	11 (23.9%)	.520
Catastrophic	4 (21.1%)	2 (16.7%)	6 (54.5%)	0 (0%)	12 (26.1%)	.077

Abbreviations: CT, calcaneotibial; PDS, polydioxanone; TESF, transarticular external skeletal fixator.

### 3.3 Surgery—Joint immobilization without tendon repair

A total of 11 of 89 limbs (12.3%) underwent temporary tarsocrural immobilization alone. Tarsocrural immobilization was achieved using calcaneotibial screws and cast in 4/11 limbs (36.4%), transarticular plates in 3/11 limbs (27.3%), TESF in 2/11 limbs (18.2%) and TESF with calcaneotibial screws in 2/11 limbs (18.2%). The transarticular plates consisted of three 2.7/3.5‐mm pantarsal arthrodesis plates.

### 3.4 Surgery—Pantarsal arthrodesis

Pantarsal arthrodesis was performed as the initial treatment choice in 32/89 limbs (36%). Medial plates were used in 20/32 limbs (62.5%), lateral plates in 7/32 limbs (21.9%) and dorsal plates in 1/32 limbs (3.1%). Plate position was unknown in 4/32 limbs (12.5%). A total of 27 of 32 plates (84.4%) were 2.7/3.5‐mm, a single plate (3.1%) was 2.0/2.7‐mm, a single plate (3.1%) was 2.7/3.5/4.5‐mm and plate size was unknown in three plates (9.4%). Calcaneotibial screws were used to augment the plates in 24/32 limbs (68.6%).

### 3.5 Postoperative management

Casts were placed postoperatively in 24/89 limbs (27%). In the tendon repair and tarsocrural immobilization group 18/46 limbs (39.1%) had calcaneotibial screws with casts and 2/46 had casts alone (4.3%). In the tarsocrural immobilization without tendon repair group 4/11 limbs (36.3%) had calcaneotibial screws and casts. A total of 13 of the 24 casts were bivalved casts (54.2%), nine were full casts (37.5%) and two were half casts (8.3%). Median duration of cast placement was 6 weeks (range: 2–8 weeks).

In the joint immobilization and tendon repair group the tarsocrural joint was immobilized for a median of 6 weeks with calcaneotibial screws and casts (range: 5–8 weeks), 6 weeks with TESF (range: 5–8 weeks) and 6 weeks with transarticular plates (range: 6–8 weeks). The two limbs with casts alone were immobilized for 6 and 8 weeks. The limb with a calcaneotibial screw alone was immobilized for 6 weeks.

When joint immobilization without tendon repair was performed the tarsocrural joint was immobilized for 6 to 8 weeks with calcaneotibial screws and casts, 6 weeks with TESF and 6 weeks with TESF and calcaneotibial screws.

The dogs were cage rested for a median of 6 weeks (range: 5–10 weeks) before a gradual return to exercise.

### 3.6 Postoperative complications

A total of 46 of 89 limbs (51.7%) experienced postoperative complications, five limbs (5.6%) had multiple complications. Six complications were minor (6.7%), seven were major type 1 (7.9%), 24 were major type 2 (26.9%) and 14 were catastrophic (15.7%). A total of 43 of 51 complications (84.3%) occurred in the perioperative period.

### 3.7 Postoperative complications—Joint immobilization with tendon repair

In the tendon repair and tarsocrural immobilization group complications occurred in 26/46 limbs (56.5%), the most common complication was infection which occurred in 12/46 limbs (26.1%). There were 12/46 catastrophic complications (26.1%) which resulted in six limbs having subsequent pantarsal arthrodesis, four limbs with recurrent plantigrade stance, one limb undergoing second tendon repair surgery and one limb amputation. The complication rate for PDS sutures was 11/19 (58.9%), and for Prolene sutures was 10/18 (55.6%). Multiple complications occurred in two limbs, one limb had minor postoperative swelling in the perioperative period and then a plantigrade stance 6 months postoperatively. The second limb had a cast related infection of the skin over the metatarsals in the perioperative period and then a failure of the CCT repair 1 year after surgery, which was managed with pantarsal arthrodesis.

When calcaneotibial screws and casts were used as the tarsocrural immobilization method the complication rate was 7/18 (38.8%) (Table 3), with four of these complications linked to the cast. Two calcaneotibial screws broke while within a cast. When TESF was used for tarsocrural immobilization the complication rate was 14/20 (70%). Nine complications were linked to the TESF, either pin tract infections or fractured metatarsals. Two of five limbs with transarticular plates had postoperative complications (40%): one limb with infection and one with screw back out.

**TABLE 3:** Complication rate for tarsocrural immobilization with tendon repair when using calcaneotibial screws and casts, TESF and transarticular plates. Multiple complications occurred in one limb in the CT screw and cast group and one limb in the transarticular plate group.

	CT screw and cast (18)	TESF (20)	Transarticular plate (5)	*p*‐value
Complication rate	7 (38.9%)	14 (70.0%)	2 (40.0%)	.129
Minor	1 (5.6%)	0 (0%)	1 (20.0%)	.160
Major type 1	1 (5.6%)	2 (10.0%)	0 (0%)	.700
Major type 2	3 (16.7%)	6 (30.0%)	1 (20.0%)	.614
Catastrophic	3 (16.7%)	6 (30.0%)	1 (20.0%)	.614

Abbreviations: CT, calcaneotibial; TESF, transarticular external skeletal fixator.

Both limbs with casts as the sole method of tarsocrural immobilization had catastrophic complications. One had tendon dehiscence with subsequent plantigrade stance. This case was recommended pantarsal arthrodesis but was lost to follow‐up. The other dog developed plantigrade stance 12 weeks after surgery and underwent subsequent pantarsal arthrodesis.

### 3.8 Postoperative complications—Joint immobilization without tendon repair

When tarsocrural immobilization was used alone complications occurred in 5/11 limbs (45.5%) (Table 4). One limb had multiple complications: a cast sore in the perioperative period and then a plantigrade stance 1 year after surgery. This case subsequently underwent pantarsal arthrodesis. The complication rate when calcaneotibial screws and casts were used was 3/4 (75%). Two complications were cast related. Two limbs had catastrophic complications which resulted in one limb having a subsequent tendon repair surgery and the other a pantarsal arthrodesis. The complication rates for TESF and TESF with calcaneotibial screws were both 1/2 (50%).

**TABLE 4:** Complication rate for tarsocrural immobilization without tendon repair when using calcaneotibial screws and casts, TESF, calcaneotibial screws and TESF and transarticular plates. Two complications occurred in one limb in the CT screw and cast group.

	CT screw and cast (4)	TESF (2)	Transarticular plate (3)	CT screw and TESF (2)	*p*‐value
Complication rate	3 (75%)	1 (50%)	0 (0%)	1 (50%)	.268
Minor	2 (50%)	0 (0%)	0 (0%)	0 (0%)	.233
Major type 1	0 (0%)	0 (0%)	0 (0%)	0 (0%)	1.000
Major type 2	0 (0%)	1 (50%)	0 (0%)	1 (50%)	.414
Catastrophic	2 (50%)	0 (0%)	0 (0%)	0 (0%)	.614

Abbreviations: CT, calcaneotibial; TESF, transarticular external skeletal fixator.

### 3.9 Postoperative complications—Pantarsal arthrodesis

The complication rate for pantarsal arthrodesis was 15/32 (46.8%), two limbs developed multiple complications. One fractured two metatarsal bones in the perioperative period and developed a bandage sore following the second surgery. The other had a surgical site infection in the perioperative period and then was found to have a broken calcaneotibial screw which required removal.

The most common complication was infection, occurring in 11/32 (34.4%) limbs, followed by fractured metatarsal bones in 2/32 limbs (6.2%), broken screws in 2/32 limbs (6.2%) and severe lameness in 1/32 limbs (3.1%).

### 3.10 Postoperative complications—Statistics

There was no difference in complication rate between tendon repair with tarsocrural immobilization and pantarsal arthrodesis (*p* = .543) or between tendon repair with tarsocrural immobilization and tarsocrural immobilization alone (*p* = 1.000). There was a higher catastrophic complication rate in the tarsocrural immobilization with tendon repair group when compared to pantarsal arthrodesis (*p* = .005). There was no difference in catastrophic complication rate between the tarsocrural immobilization without tendon repair group and the pantarsal arthrodesis group (*p* = .061). The use of three‐loop pulley sutures or locking loop sutures did not impact complication rate (*p* = 1.000). There was no difference in complication rate between PDS and Prolene (*p* = 1.000).

### 3.11 Short‐term outcomes

Outcome was categorized in 85 limbs at final follow‐up and median time from surgery to final follow‐up at the referral center was 10 weeks (range: 6–256 weeks). A total of 56 of 89 limbs had a follow‐up of <12 weeks (62.9%), 14/89 (15.7%) limbs had a follow‐up of 12 to 25 weeks, 9/89 limbs had a follow‐up of 25–52 weeks (10.1%) and 10/89 limbs had a follow‐up of more than 52 weeks (11.2%). Median follow‐up time for tarsocrural immobilization with tendon repair was 8 weeks (range: 6–260), for pantarsal arthrodesis was 13 weeks (range: 6–204) and for tarsocrural immobilization without tendon repair was 9 weeks (range: 6–134). Outcome at the final assessment for the initial surgical treatment option was categorized as full function in 21/89 limbs (23.6%), acceptable function in 43/89 limbs (48.3%) and unacceptable function in 21/89 limbs (23.6%). Outcome was unknown in 4/89 limbs (4.5%). There was no difference in unacceptable function rates between acute and chronic cases (*p* = .758).

Of the six dogs that underwent subsequent pantarsal arthrodesis: five had acceptable function at follow‐up examination and one had unacceptable function due to persistent lameness. The two limbs that had a second tendon repair surgery both had acceptable function at follow‐up examination.

The postoperative lameness score was recorded in 58/89 (65.2%) limbs and median postoperative lameness score was 1/5 (range: 0/5–5/5) for pantarsal arthrodesis, 3/5 (range: 0/5–5/5) for tendon repair with temporary tarsocrural immobilization and 3/5 (range: 0/5–5/5) for temporary tarsocrural immobilization without tendon repair. There was a decrease in median lameness score for pantarsal arthrodesis (*p* = .005) but not for repair with tarsocrural immobilization (*p* = 1.000) or tarsocrural immobilization without tendon repair (*p* = .253).

### 3.12 Questionnaires

A total of 23 LOAD questionnaires were returned by owners (26.9%) (Table 5). One case underwent staged bilateral procedures, three‐loop pulley and locking loop sutures alongside TESF were used as the method of tendon repair and tarsocrural immobilization in both limbs. This case had a LOAD score of 18. Median time from surgery to completion of the questionnaire was 48 months (range 10–109 months). Two of 24 questionnaires (8.3%) were returned within 1 year of surgery, 14/24 (58.3%) between 2 and 5 years, and 8/24 (33.3%) between 5 and 10 years following surgery.

**TABLE 5:** LOAD questionnaire groups.

Questionnaire results	Tendon repair with tarsocrural immobilization	Pantarsal arthrodesis	Tarsocrural immobilization without tendon repair	Total	*p*‐value
Total questionnaires returned	15 (62.5%)	3 (12.5%)	6 (25%)	24 (100%)	
Mildly affected	5 (33.3%)	1 (33.3%)	4 (66.7%)	10 (41.7%)	.493
Moderately affected	7 (46.7%)	0 (0%)	2 (33.3%)	9 (37.5%)	.421
Severely affected	2 (13.3%)	2 (66.7%)	0 (0%)	4 (16.7%)	.097
Extremely affected	1 (6.7%)	0 (0%)	0 (0%)	1 (4.2%)	1.000

Abbreviation: LOAD, Liverpool Osteoarthritis in Dogs.

## 4 DISCUSSION

This study reports the surgical procedures, complications and outcomes of 89 surgeries performed for CCT pathology. Our hypothesis that pantarsal arthrodesis as the initial surgical treatment for Achilles tendon pathology would reduce complication rates and improve outcomes was only partially supported. While there was no difference in overall complications or outcomes between the three surgical groups, pantarsal arthrodesis was associated with a significantly lower rate of catastrophic complications compared to tendon repair with tarsocrural immobilization.

Consistent with previous studies the majority of dogs were medium to large breed and Labrador Retrievers were overrepresented.^3^, ^4^, ^18^, ^20^ In contrast, Dobermans were underrepresented with only three dogs (3.8%), this likely reflects a decreasing popularity of Dobermans rather than a change to the breed.^27^ There appears to be no gender predisposition for CCT pathology in dogs, with this study and others showing no gender bias for the development of degenerative CCT pathology.^4^ In humans there is conflicting evidence for gender being a risk factor for development of common calcaneal tendinopathy, with different studies reporting being male or female as a risk factor.^3^, ^4^, ^28^

Suture patterns that resist gap formation are essential for CCT repair as, despite tarsocrural immobilization, the tendon remains under strain due to contraction of the muscles of the CCT mechanism.^29^ Both this and previous retrospective studies have reported no difference in clinical complication rate between three‐loop pulley and locking loop sutures.^4^ Three‐loop pulley and locking loop sutures appear suitable for CCT repair if protected through tarsocrural immobilization, as they are able to resist gap formation under the strain experienced while the tarsocrural joint is immobilized.

As with previous studies the complication rate for calcaneotibial screws with casts was lower than TESF, although in the current study this did not reach significance there was a trend toward significance.^4^ It is widely reported that the majority of postoperative complications following CCT surgery are related to the tarsocrural immobilization method.^4^, ^20^ This is reflected in the current study as 13/21 complications in the tendon repair group were either pin tract infections or cast related. In two limbs the calcaneotibial screw broke while in a cast, suggesting that cast did not protect the screw from strain during loading. There are however several factors, such as screw angle and size, that must be considered as possible reasons for calcaneotibial screw failure.^30^ Only a single case in this study had a calcaneotibial screw without a cast, allowing no meaningful comparisons to be made. Using calcaneotibial screws alone may reduce postoperative complication rates, however, further studies are needed to compare calcaneotibial screws both with and without casts.

Both limbs with casts as the sole method of tarsocrural immobilization following surgery had catastrophic complications. It is unclear as to why both limbs suffered catastrophic complications as previous studies have reported no difference in complications between casts/splints alone and TESF, or only minor cast related complications.^4^, ^19^, ^20^

Transarticular plates offer an alternative method of temporary tarsocrural immobilization with both calcaneotibial and tibiometatarsal plates being used, following CCT repair surgery, with low complication rates and good outcomes.^19^, ^31^ The use of a minimally invasive approach when placing transarticular plates reduces patient trauma and the comorbidities seen with TESF and casts are avoided. While reported use of transarticular plates is low, they may provide the stabilization required to protect the CCT during healing with a lower postoperative complication rate than alternative tarsocrural immobilization methods.

Catastrophic complications were more common following tendon repair and tarsocrural immobilization compared to pantarsal arthrodesis. Additionally, temporary tarsocrural immobilization without tendon repair exhibited a complication rate that trended toward significance when compared to arthrodesis. This must be taken into account when choosing a surgical option and discussing them with clients. However, one must consider that catastrophic complications of these surgeries can still be managed with pantarsal arthrodesis, whereas following pantarsal arthrodesis the options include revision surgery, amputation or euthanasia. This may create a bias in the distribution of complications. The catastrophic complication rate for pantarsal arthrodesis is also lower in comparison to other studies.^23^ Degenerative CCT pathology without diseases or injuries involving the joints of the tarsus means that there is likely a superior environment for bone fusion resulting in a lower complication rate. Function of the hock is lost with pantarsal arthrodesis, however when long term outcomes have been assessed through owner completed questionnaires, similar rates of satisfaction are reported for pantarsal arthrodesis and for CCT repair surgery.^4^, ^23^

Short‐term outcomes favored pantarsal arthrodesis, with a lower unacceptable function rate than tendon repair, with rates of 14% and 30%, respectively, this, however, did not reach significance (*p* = .205). The higher rate of unacceptable function and higher postoperative lameness scores in the tendon repair group may be due to tendon repair cases taking longer to return to expected function than pantarsal arthrodesis. Neilson et al. reported the mean time to best function following CCT repair was 20 weeks, whereas in the current study the median time from surgery to last in clinic examination was 8 weeks.^20^ With a longer period from surgery to final examination the function of the CCT repair surgeries may have improved and lameness scores decreased. This is partially observed in the LOAD scores, where in the tendon repair and tarsocrural immobilization group 80% of limbs were mildly and moderately affected compared to 33.3% in the pantarsal arthrodesis group. These figures should be taken with caution due to the low number of questionnaires returned, particularly for pantarsal arthrodesis, and the difference not reaching significance.

The limitations of this study are that it is retrospective, resulting in a wide range in surgical techniques, missing data and a lack of information as to why clinical decisions were made. Radiographs could not be identified for every case which may have resulted in missed complications. Details of imaging findings and the exact locations of tendon injury were inconsistently reported and subsequently were not analyzed. Both factors may contribute to clinical decision making and outcome of CCT repair and should be included in future studies. There is no quantitative data on limb function following surgery and force plate analysis would provide a useful comparison on limb use between pantarsal arthrodesis, tendon repair with immobilization and immobilization without repair.

Additionally, the overall follow‐up time in the medical records was relatively short, limiting the ability to detect later developing complications. There was a low number of questionnaires returned. This was partly due to the long period of time from which cases were obtained, and many patients were deceased before their owners could be contacted. There is also the potential for a non‐responder bias when collecting a questionnaire. This study's findings should be interpreted with consideration of potential type I and type II errors. Small sample sizes in subgroups, particularly the tarsocrural immobilization with tendon repair cohort, reduced statistical power, increasing the risk of type II errors. Conversely, the use of a standard significance threshold (*p* = .05) may increase the risk of type I errors due to multiple comparisons. Future studies with larger cohorts are needed to mitigate these limitations and validate observed associations.

Despite the limitations of this study, our findings indicate a greater risk of complications requiring revision surgery following temporary tarsocrural immobilization, with or without tendon repair, compared to pantarsal arthrodesis. While preserving normal hock function is often a clinical priority, the increased risk of revision surgery should be discussed with owners, considering the potential financial and treatment implications for their dogs.

## AUTHOR CONTRIBUTIONS

Wylie S, BVetMed, PGDipVCP, FHEA, MRCVS: Identified suitable medical records, recorded demographic information, compiled all data, contacted owners for questionnaire completion, interpreted data, analyzed data for statistical significance, drafted and revised the manuscript. Collected retrospective data for 27 cases, LOAD questionnaires for nine cases. Piana F, DVM(Hons), PGDipVCP, DipECVS, MRCVS: Contributed to study design, identified suitable medical records, data interpretation, in‐line editing of manuscript. Montgomery P, BMVS, CertAVP(GSAS), PGCert(VPS), MRCVS: Identified suitable medical records, contacted owners for questionnaire completion, compiled data. Collected retrospective data for 24 cases, LOAD questionnaires for three cases. Girling S, BVSc, CertSAS, DipECVS, MRCVS: Identified suitable medical records, compiled data, provided in‐line editing of the manuscript. Collected retrospective data for six cases. Vezzoni L, DVM, DipECVS: Identified suitable medical records, contacted owners for questionnaire completion, compiled data, provided in‐line editing of the manuscript. Collected retrospective data for six cases, LOAD questionnaires for six cases. Meeson R, MA, VetMB, PhD, MVetMed, DipECVS, FHEA, FRCVS: Identified suitable medical records, compiled data, provided in‐line editing of the manuscript. Collected retrospective data for 18 cases, LOAD questionnaires for five cases. Belch A, BVMS, MSc, CertAVP(GSAS), DipECVS, MRCVS: Contributed to data interpretation, in‐line editing of manuscript. Parsons K, BVSc (Hons), PhD, DipECVS, FHEA, FRCVS: Contributed to study design, interpreted data, provided in‐line editing of the manuscript.

## FUNDING INFORMATION

No funding was received for this research.

## CONFLICT OF INTEREST STATEMENT

The authors have no conflicts of interest to disclose.
